# Comprehensive liquid biopsy analysis for monitoring NSCLC patients under second-line osimertinib treatment

**DOI:** 10.3389/fonc.2024.1435537

**Published:** 2024-10-21

**Authors:** Aliki Ntzifa, Theodoros Marras, Galatea Kallergi, Athanasios Kotsakis, Vasilis Georgoulias, Evi Lianidou

**Affiliations:** ^1^ Analysis of Circulating Tumor Cells Lab, Lab of Analytical Chemistry, Department of Chemistry, National and Kapodistrian University of Athens, Athens, Greece; ^2^ Laboratory of Biochemistry/Metastatic Signaling, Section of Genetics, Cell Biology and Development, Department of Biology, University of Patras, Patras, Greece; ^3^ Department of Medical Oncology, General University Hospital of Larissa, Larissa, Greece; ^4^ First Department of Medical Oncology, Metropolitan General Hospital of Athens, Cholargos, Greece

**Keywords:** NSCLC, osimertinib, liquid biopsy, ctDNA, CTC, resistance

## Abstract

**Background:**

The heterogeneous and complex genetic landscape of NSCLC impacts the clinical outcomes of patients who will eventually develop resistance to osimertinib. Liquid biopsy (LB) analysis as a minimally invasive approach is a key step to efficiently identify resistance mechanisms and adjust to proper subsequent treatments.

**Materials and methods:**

In the present study, we combined plasma-cfDNA and CTC analysis from 30 NSCLC patients in samples collected before treatment and at the progression of disease (PD). We detected molecular alterations at the DNA mutation (*EGFR, PIK3CA, KRAS* G12C, *BRAF* V600E), DNA methylation (*RASSF1A, BRMS1, FOXA1, SLFN1, SHISA3, RARβ,, WIF-1, RASSF10* and *APC*), gene expression (*CK-19, CK-18, CK-8, AXL, TWIST-1, PD-L1, PIM-1, Vimentin, ALDH-1*, and *B2M*) and chromosomal level (*HER2* and *MET* amplification) as possible resistance mechanisms and druggable targets. We also studied the expression of PD-L1 in single CTCs using immunofluorescence.

**Results:**

In some cases, T790M resistance *EGFR* mutation was detected at baseline in CTCs but not in the corresponding plasma cfDNA. *PIK3CA* mutations were detected only in plasma-cfDNA but not in corresponding CTCs. *KRAS* G12C and *BRAF* V600E mutations were not detected in the samples analyzed. *MET* amplification was detected in the CTCs of two patients before treatment whereas *HER2* amplification was detected in the CTCs of three patients at baseline and in one patient at PD. DNA methylation analysis revealed low concordance between CTCs and cfDNA, indicating the complementary information obtained through parallel LB analysis. Results from gene expression analysis indicated high rates of vimentin-positive CTCs detected at all time points during osimertinib. Moreover, there was an increased number of NSCLC patients at PD harboring CTCs positive in *PD-L1. AXL* and *PIM-1* expression detected in CTCs during treatment suggesting new possible therapeutic strategies.

**Discussion:**

Our results reveal that comprehensive liquid biopsy analysis can efficiently represent the heterogeneous molecular landscape and provide prominent information on subsequent treatments for NSCLC patients at PD since druggable molecular alterations were detected in CTCs.

## 1 Introduction

During the last twenty years, the emergence of molecular targeted therapies has significantly changed non-small cell lung cancer (NSCLC) treatments since it was shown that they are more effective over chemotherapeutic regimens (1, 2). Several clinical trials have clearly demonstrated that tyrosine kinase inhibitors (TKI) of the epidermal growth factor receptor (EGFR) have achieved improved clinical outcomes for EGFR mutant (EGFRm) NSCLC patients (3–6). Osimertinib, a third-generation EGFR TKI, has changed the therapeutic management of NSCLC patients (7, 8). Initially, only EGFRm NSCLC patients that were previously treated with first- or second-generation EGFR TKIs (9–12) were subjected to osimertinib treatment. Still, this drug is now the standard of care for first-line therapy (13, 14). Recently osimertinib has been administered as an adjuvant treatment since clinical outcomes are significantly improved (15–17). The clinical benefit of this drug is now being investigated in the neoadjuvant setting (18, 19).

Acquisition of new mutations or pre-existing genetic alterations is linked to disease progression in NSCLC patients with *EGFR* mutations (20–23), and the highly heterogeneous and complex genetic landscape of this type of cancer impacts clinical outcomes. This is a major reason for the emergence of resistance mechanisms to this type of therapy in these patients (24–26). The most common resistance mechanism to first- and second-generation EGFR TKIs is the T790M EGFR mutation that is now therapeutically targeted by osimertinib (27). The heterogeneity of resistance mechanisms is the main reason that it is such a difficult challenge to overcome resistance to osimertinib (28). These acquired resistance mechanisms can be either *EGFR*-dependent or *EGFR*-independent (25, 29). We now know that activation of MET or HER2 amplification, acquisition of mutations in *BRAF*, *PIK3CA*, and *KRAS*, histological transformation to small cell lung cancer (SCLC), and epithelial-to-mesenchymal transition (EMT) are major reasons (30–35). Results from the AURA clinical trials underlined the use of ctDNA analysis in reflecting tumor heterogeneity, monitoring the efficacy of EGFR TKIs as well as the early detection of resistance mechanisms (7, 8, 10, 36).

The key step to efficiently overcome resistance mechanisms and adjust to proper subsequent treatments is to identify the resistance much earlier than conventional strategies (37). Liquid biopsy (LB) analysis as a minimally invasive approach to longitudinally and regularly monitoring NSCLC patients plays a pivotal role in early tracking tumor evolution and relapse (1, 28, 38). The increasing number of LB tests that have been cleared by the US Food and Drug Administration (FDA) has led to the application of circulating tumor DNA (ctDNA) analysis in clinical routine testing in NSCLC) (39), and recent recommendations suggest these tests for patients with advanced or metastatic NSCLC especially when tissue sampling cannot be performed (40–46). A recent comprehensive genomic profiling (CGP) of osimertinib resistance mechanisms performed in primary tumors or peripheral blood of NSCLC patients concluded that this type of analysis could help select therapies (47). Conversely, many studies have shown that circulating tumor cell (CTC) analysis in EGFRm NSCLC could contribute to the management of patients in a complementary way (48–52). It has been clearly shown that CTC analysis gives significant information on tumor heterogeneity and clonal evolution occurring under treatment (48–51, 53). Thus, CTC analysis would aid in adjusting targeted therapy for EGFRm NSCLC patients based on the resistance mechanisms identified.

It is now evident that CTCs when compared to ctDNA provide complementary information, thus comprehensive LB analysis is highly essential for the management of cancer patients. Moreover, CTCs better depict tumor heterogeneity and provide unique information derived from many different cellular components that cannot be revealed by ctDNA analysis (54). Lately, few but still important studies have shown the clinical significance of combining the information derived from different LB analytes in various types of cancer, such as breast (55–61), NSCLC (49, 62, 63), prostate (64), and very recently in melanoma (65). Regarding the treatment monitoring of NSCLC patients, a comprehensive analysis of *EGFR* mutations in cfDNA and CTCs could be more informative as this was recently demonstrated in studies that included both LB analytes (66, 67). Besides, it has already been demonstrated that several distinct molecular features contribute to the heterogeneity of NSCLC apart from *EGFR* tumor clonality that arises during cancer development and treatment (21). Tumor clonality in conjunction with the highly heterogeneous landscape of resistance in NSCLC patients under osimertinib claims for the identification of new molecular biomarkers that could be potential therapeutic targets (23, 28, 68). Therefore, analyzing more than one LB analytes in patients’ samples as an integrated approach could be more beneficial for the early detection of resistance and efficient treatment management. Our group has extensively studied the combination of ctDNA and CTC analysis in NSCLC patients during osimertinib therapy (69–72).

In the current study, we performed a comprehensive LB analysis for monitoring NSCLC patients under second-line osimertinib treatment, by combining plasma-cfDNA and matched CTC to identify molecular alterations (DNA mutations, DNA methylation, and gene expression) as well as chromosomal alterations that could be early indicators of resistance and provide potential targets for subsequent treatments.

## 2 Materials and methods

### 2.1 Patients

Thirty patients, recruited through a multicenter Phase II clinical study [ClinicalTrials.gov number: NCT02771314], all diagnosed with EGFR mutated lung adenocarcinomas, and treated with osimertinib (AZD9291; Astra Zeneca, UK) were included. Peripheral blood from ten healthy donors (HD) was used as a control group. The study was conducted following the Declaration of Helsinki, and all patients and HD gave their written informed consent. The study was approved by the National Drug Administration of Greece (EOF), the National Ethics Committee (35/00-03/16, 35/03-11/16) and the Institutional Ethical Committees of the HORG’s participating centers.

### 2.2 Collection of peripheral blood samples

**Figure 1:**
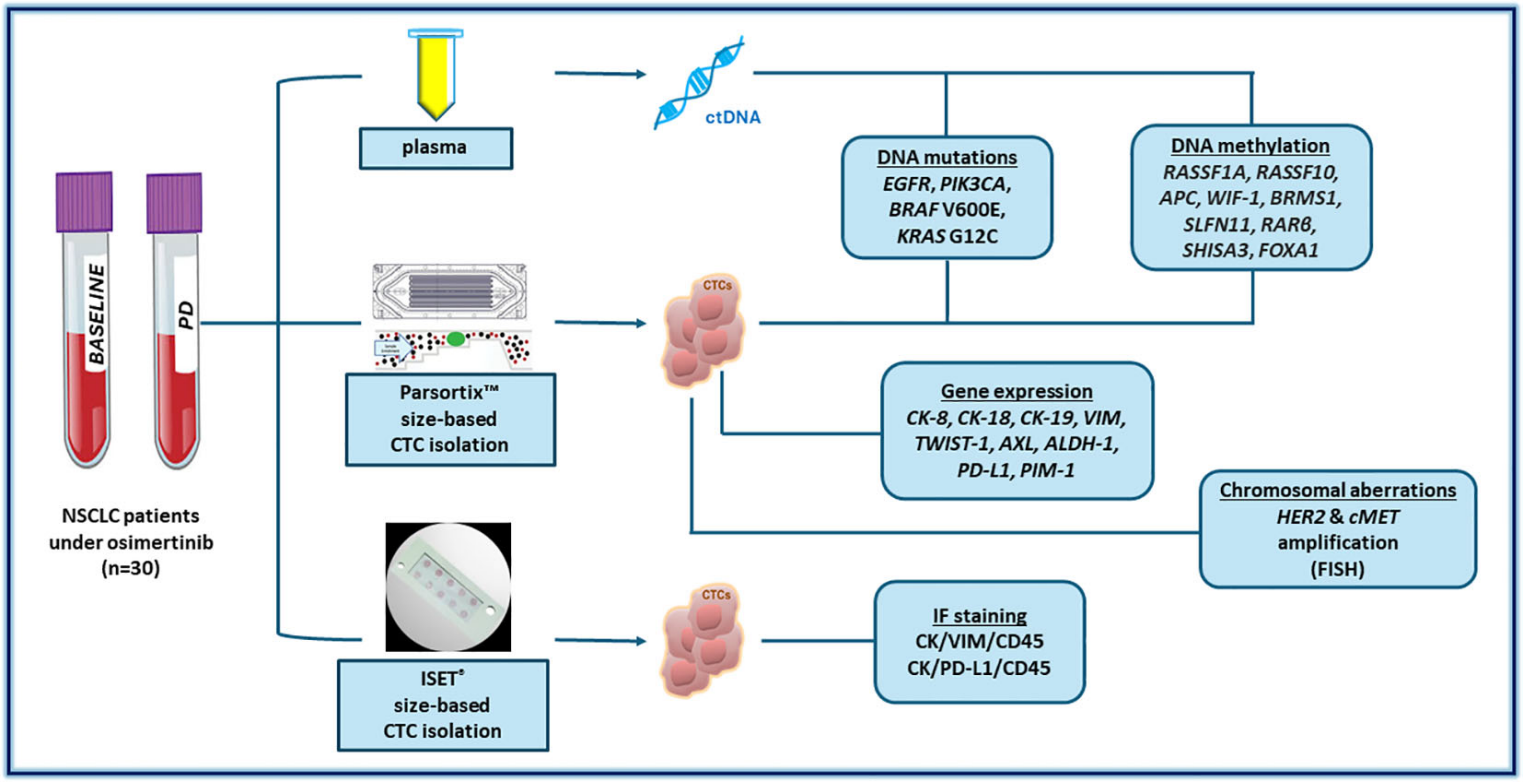
A schematic flowchart of the study.

Peripheral blood (PB) was collected at baseline before treatment initiation and at disease progression (PD). PB (30mL) was collected in EDTA tubes and the first 5mL of blood was discarded to avoid contamination of skin epithelial cells. Plasma separation from buffy coat and erythrocytes was performed as previously described (69, 70, 73) (**Figure 1**).

### 2.3 Plasma-cfDNA extraction and CTC enrichment

For each blood draw, plasma samples were aliquoted; 2.00 mL plasma was used for cfDNA isolation, using silica-based membrane extraction kits: cobas^®^ cfDNA Sample Preparation Kit (Roche Molecular Systems, Inc.) for downstream EGFR mutation analysis. Another aliquot of 2.00 mL plasma was used for cfDNA isolation for downstream Crystal Digital PCR analysis with the naica® system (Stilla Technologies, Villejuif, France) (70). Another identical plasma aliquot was used for cfDNA extraction for downstream mutation analysis of *PIK3CA, KRAS*, and *BRAF*, as reported below, using the QIAamp Circulating Nucleic Acid Kit (Qiagen^®^, Hilden, Germany) was used.

Following plasma separation, CTC enrichment was performed, using the FDA-cleared Parsortix™ (ANGLE plc, UK) device (73), and the CTC-enriched fraction was collected in 200 μL of PBS. Total RNA was extracted from the CTC-enriched fraction using TRIZOL followed by cDNA synthesis (73). Genomic DNA (gDNA) was extracted from the CTC-enriched fraction using TRIZOL as previously described (70). Ten mL of peripheral blood in EDTA using identical blood draws was used for CTC enrichment with ISET (Rarecells Diagnostics, France, and downstream molecular characterization by triple immunofluorescence.

### 2.4 Whole genome amplification

Whole genome amplification of gDNA extracted from the enriched CTCs was performed using a commercially available kit (Ampli1™ Whole Genome Amplification, Menarini Silicon Biosystems, Italy) (70).

### 2.5 Plasma-cfDNA and paired CTC analysis: DNA mutations

#### 2.5.1 *EGFR* mutations

All plasma-cfDNA samples were analyzed by the FDA-cleared cobas^®^ EGFR Mutation Test v2 in the cobas^®^ z 480 analyzer (Roche) (70), a test for which our lab has an ISO-15189 accreditation (74). Plasma-cfDNA isolated from identical plasma aliquots and paired CTC-derived gDNA were further analyzed by Crystal digital PCR™ as previously described (70).

#### 2.5.2 *PIK3CA* mutations

All plasma-cfDNA samples and paired CTC-derived gDNA samples were analyzed for the presence of three *PIK3CA* hotspot mutations (p.E545K exon 9, p.E542K exon 9 and p.H1047R exon 20) using our previously described ultrasensitive real-time PCR methodology (75).

#### 2.5.3 *KRAS* G12C and *BRAF* V600E

Droplet digital PCR (ddPCR) was used for the analysis of cfDNA and paired CTC-derived gDNA samples for *KRAS* G12C and *BRAF* V600E mutations in a BioRad QX200 ddPCR System using a commercially available ddPCR reaction mix and specific primers and probes (Bio-Rad Laboratories).

### 2.6 DNA methylation analysis in plasma-cfDNA and paired CTC-derived gDNA

All cfDNA samples and paired CTC-derived gDNA samples were subjected to Sodium Bisulfite (SB) treatment as previously described (69). SB-treated samples were subsequently analyzed for the DNA methylation of *RASSF10*, *WIF-1*, *APC*, *RARβ*, *RASSF1A*, *BRMS1*, *FOXA1, SLFN1*, *SHISA3* genes with our previously developed and analytically validated real-time methylation-specific PCR (MSP) assays (69).

### 2.7 CTC analysis: gene expression

Gene expression was studied in CTC-derived total RNA by RT-qPCR for the following genes: *CK-8*, *CK-18*, *CK-19*, *Vimentin*, *TWIST-1, AXL, ALDH-1*, *PD-L1*, *PIM-1* and *B2M* as previously reported (73).

### 2.8 CTC analysis: triple immunofluorescence

CTCs captured in the ISET filters were subsequently analyzed by triple immunofluorescence for CK/VIM/CD45 using the Confocal laser Scanning microscopy (LEICA), as previously described (73). For CK/PD-L1/CD45, the process was according to our previous report (71).

### 2.9 CTC analysis: fluorescent *in situ* hybridization

#### 2.9.1 Detection of *HER2*

Amplification by FISH was performed on enriched CTCs, using PathVysion HER2 DNA Probe Kit (Abbott Molecular, Inc). FISH signal patterns were determined for the *HER2* gene and the centromere of chromosome 17 in a fluorescent microscope (Axioplan 2, Zeiss, and Leica GSL120) equipped with Cytovision Image Analysis Software. The ratio of the total number of HER-2/neu (red signals) as compared to the total number of CEP 17 (green signals) was calculated for every nucleus. When the HER2/CEP17 ratio was ≥2 a cell was considered as *HER2*-amplified according to the manufacturer and previous reports (76, 77).

#### 2.9.2 *C-MET* amplification

FISH analysis for the detection of *c-MET* amplification was performed on enriched CTCs, using c-MET (MET) Amplification Probe (Cytocell). The evaluation of *MET* amplification is based on the determination of FISH signal patterns for *MET* and the centromere of chromosome 7, in a fluorescent microscope (Axioplan 2, Zeiss and Leica GSL120) equipped with Cytovision Image Analysis Software. When the C-MET/CEP7 ratio was ≥2 the cell was considered as c-MET-amplified according to the manufacturer and previous reports (78).

## 3 Results

### 3.1 Plasma-cfDNA and paired CTC analysis: DNA gene mutations

**Figure 2:**
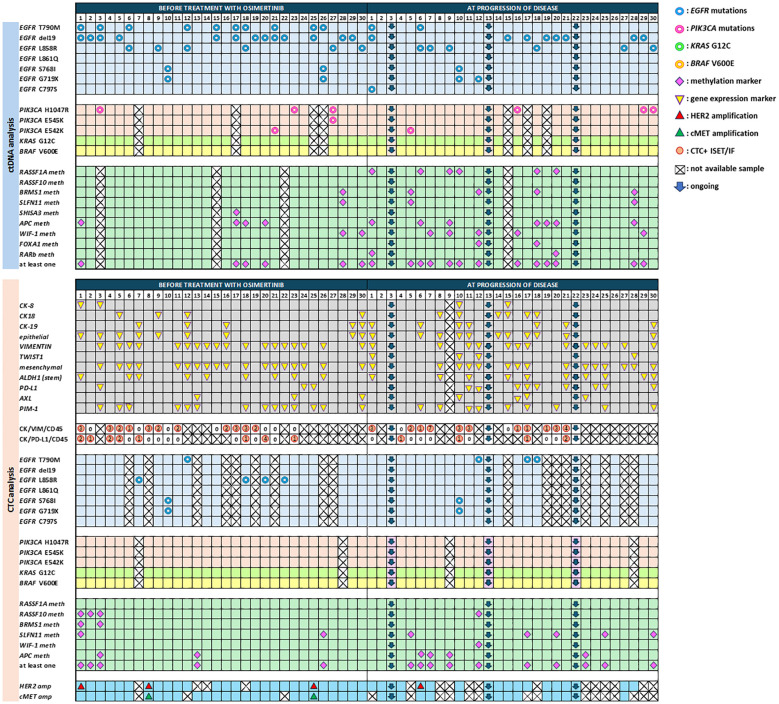
Comprehensive analysis in cfDNA and CTCs of NSCLC patients before treatment with osimertinib and at progression of disease.

We have previously analyzed plasma-cfDNA samples for the detection of *EGFR* mutations both with crystal dPCR and with the FDA-cleared assay cobas EGFR mutation test v2 (70) and reported high concordance rates between the two methodologies. By combining the results for the subgroup of patients included in the current study, T790M mutation was detected in 10/30 (33.3%), exon19 deletions in 13/30 (44.3%), L858R in 6/30 (20%), S768I and G719X in 2/30 (6.7%) patients, at baseline. At PD, T790M was detected in 2/27 (7.4%), exon19 deletions in 8/27 (29.6%), L858R in 6/27 (22.2%), S768I in 1/27 (3.7%) and G719X in 2/27 (7.4%) (**Figure 2**). In addition, C797S was found *in trans* configuration with T790M in patient #1 (P#1) at PD. In parallel, we have also analyzed CTC-gDNA samples for *EGFR* mutations using crystal dPCR. In baseline, one patient was found positive for T790M and 4/21 (19%) patients for L858R. P#10 was found positive for S768I and G719X mutations both in baseline and PD samples. T790M was detected only in 3/19 (15.8%) CTC-gDNA samples at PD (**Figure 2**).

In this study, we have analyzed plasma-cfDNA samples (n=50) and paired CTC-gDNA samples (n=53) for the detection of *PIK3CA* mutations (H1047R, E542K, E545K). at two time points. *PIK3CA* mutations were detected in 4/26 (15.4%) cfDNA samples at baseline, and 4/24 (16.7%) at PD. More precisely, H1047R was found only in the baseline samples of three patients (P#3, P#23, P#27), E545K was found concomitantly with H1047R in patient P#27, and E542K was found in P#21 (**Figure 2**). At PD, H1047R has emerged in three patients (P#16, P#29, P#30), E542K was found only in P#5, and E545K was not detected in any sample. In CTC-gDNA samples, no *PIK3CA* mutations were detected at any time point (**Figure 2**). When these 50 plasma-cfDNA and 53 paired CTC-derived gDNA samples were analyzed for the detection of *KRAS* G12C and *BRAF* V600E mutations with ddPCR, they were all found negative for both mutations.

### 3.2 Plasma-cfDNA and paired CTC analysis: DNA methylation

At baseline one methylation marker was detected at least in 6/27 (22.2%) patients at PD in 13/26 (50%). Even if methylation for these markers was detected in more samples at PD in respect to baseline there was no statistically significant difference (**Figure 2**). CTC analysis also revealed an overall increase in the detection of DNA methylation markers at PD. More specifically, at baseline, 5/30 (16.7%) samples and at PD 10/27 (37%) were found positive for at least one methylation marker (**Figure 2**).

### 3.3 CTC analysis: gene expression

In CTC-enriched fractions before treatment, 9/30 (30%) patients were positive for at least one epithelial marker in CTCs (*CK-8*, and/or *CK-18*, and/or *CK-19*) whereas at PD more samples were positive for the expression of epithelial markers (**Figure 2**), but there was no statistically significant difference between these two time points. The same was seen for the mesenchymal/EMT markers tested (*VIM, AXL*, and *TWIST-1)* [18/30 (60%) versus 15/26 (57.7%)], for *ALDH*-*1*, that was detected in 9/30 (30%) samples at baseline and 10/26 (38.5%) at PD and for *PIM-1* overexpression that was detected in 14/30 (46.7%) baseline samples and 9/26 (34.6%) at PD. *PD-L1* expression levels were significantly different between baseline and disease progression [3/30(10%) patients versus 9/26 (34.6%), McNemar test, *p* = 0.016], as this was previously described (73), (**Figure 2**).

### 3.4 CTC analysis: characterization of ISET-enriched CTCs by triple IF

CTC molecular characterization at the single-cell level was performed by using a combination of the size-based isolation platform, ISET, and confocal microscopy in 31 CTC samples, at baseline and PD. A direct comparison between RT-qPCR and IF staining for the presence of CTC positive for CK (CK-8, CK-18, CK-19) and/or VIM revealed an agreement of 54.8% (17/31 samples) (**Figure 2**).

**Figure 3:**
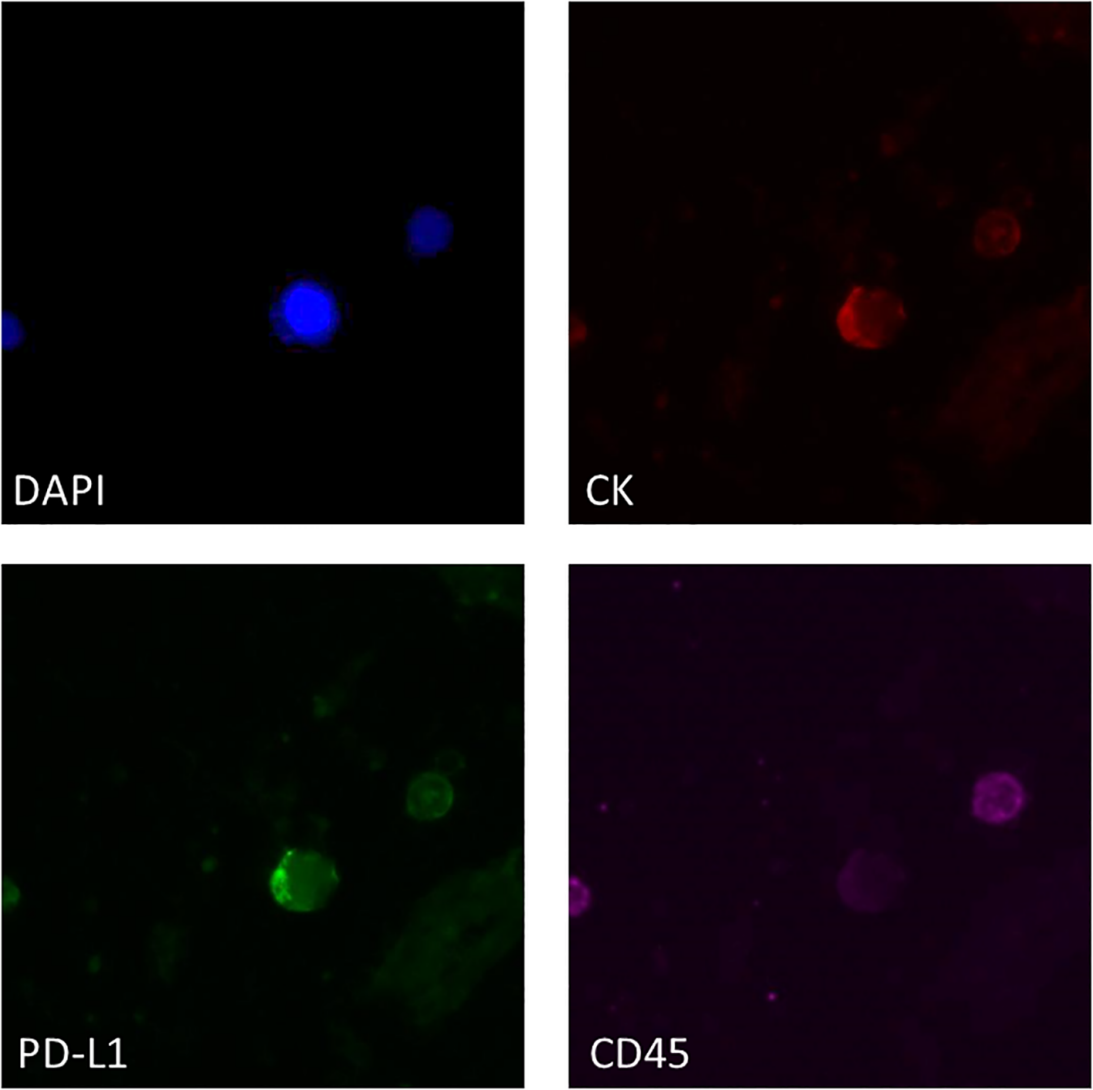
Representative IF image of PD-L1 expression in ISET-enriched CTCs. CTCs stained with CK (red), PD-L1 (green) and CD45 (purple). Nuclei (blue) were stained with DAPI.

The same ISET filters were analyzed for the phenotype CK^+^PD-L1^+^CD45^−^ in 16 baseline samples and 11 PD samples matched with those analyzed for the mRNA expression of *PD-L1* with RT-qPCR. A representative image from PD-L1 positive CTCs enriched with the ISET is shown in **Figure 3**. Combining the results from RT-qPCR and IF for the presence of *PD-L1* in CTCs, we found 5/11 (45.4%) patients at baseline and 8/17 (47%) patients at PD with CTCs and/or CTC-gDNA positive for PD-L1 (**Figure 2**).

### 3.5 CTC analysis: fluorescence *in situ* hybridization

**Figure 4:**
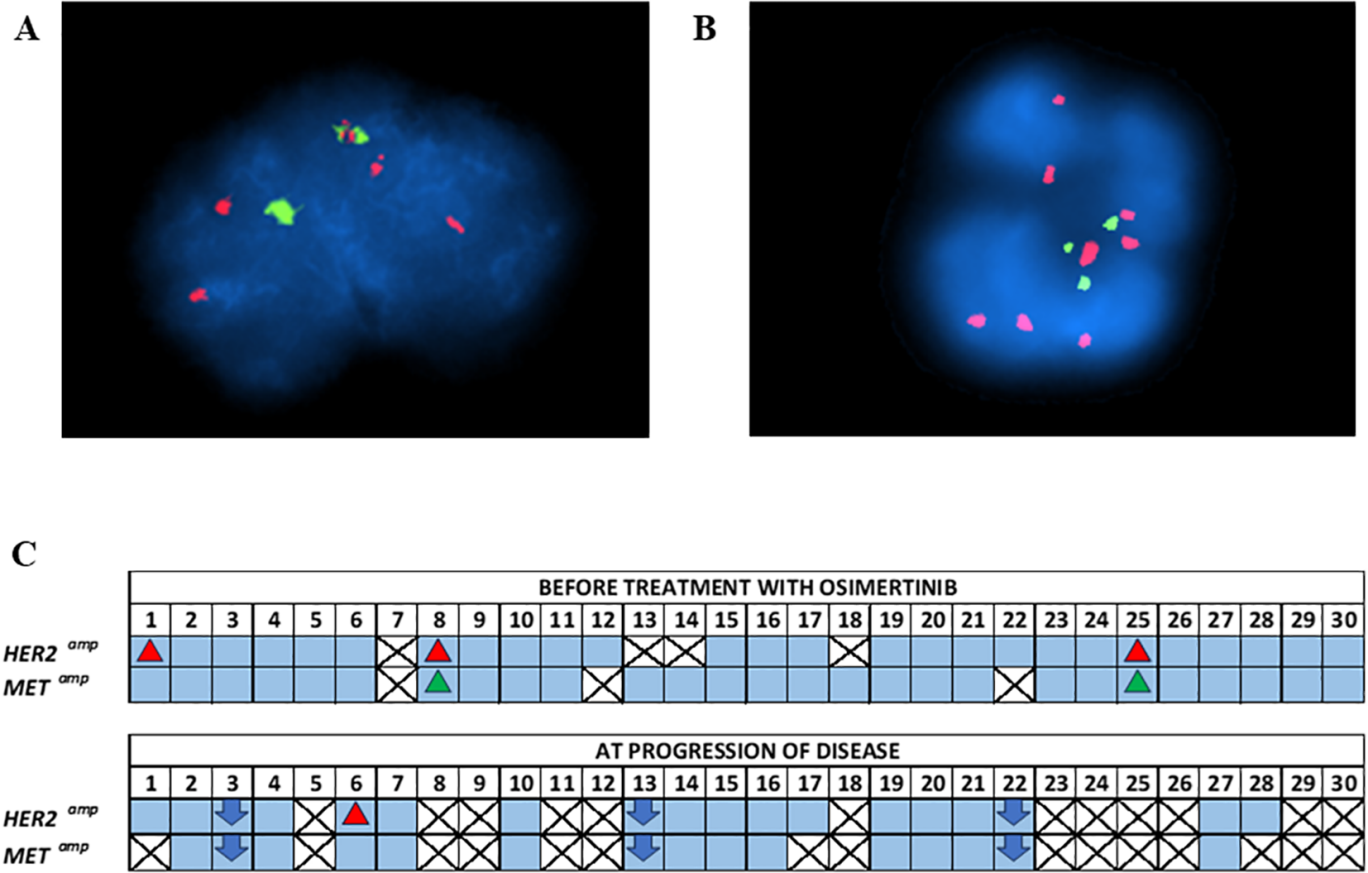
**(A)** Representative FISH image of CTC with *HER2* amplification. Red and green signals within nuclei indicate the *HER2* gene and the centromere 17 (CEP17), respectively, **(B)** Representative FISH image of CTC with *cMET* amplification. Red and green signals within nuclei indicate the *cMET* gene and the centromere 7 (CEP7), respectively, **(C)** FISH analysis of enriched CTCs of NSCLC patients under osimertinib treatment at baseline and at PD.

FISH analysis was performed on enriched CTCs from these 30 NSCLC patients for the detection of *HER2* amplification at two time points: a) baseline (n=26) and b) PD (n=15) (**Figure 4**). *HER2* amplification was found in 3/26 (11.5%) patients at baseline and 1/15 (6.7%) at PD. For the detection of *MET* amplification, FISH analysis was performed on enriched CTCs also at two time points: a) baseline (n=27) and b) PD (n=12). *MET* amplification was found in 2/27 (7.4%) patients at baseline whereas there was no positive sample at PD. Patient#8 (P#8) and P#25 had concomitant *HER2* and *MET* amplification in their enriched CTCs at baseline.

### 3.6 Molecular alterations at PD

**Figure 5:**
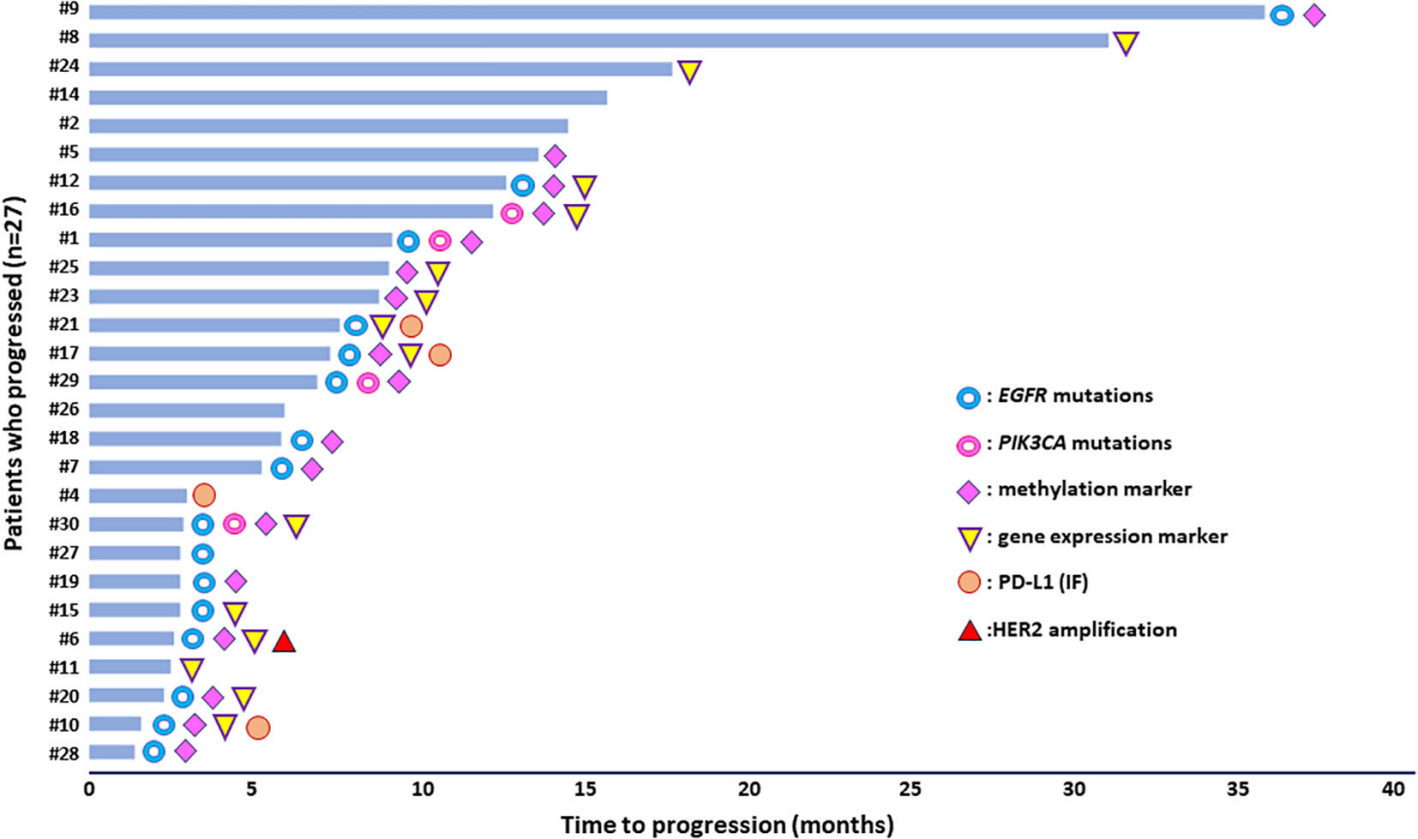
Molecular alterations detected in plasma-cfDNA and/or paired CTCs of NSCLC patients under osimertinib treatment in correlation with time to progression.

Molecular alterations detected at PD either in plasma-cfDNA samples or in paired CTCs of NSCLC patients (n=27) treated with osimertinib and who finally progressed during this analysis in DNA mutation, DNA methylation, gene expression, and chromosomal level are summarized in detail in **Table 1**. **Figure 5** depicts the presence of the molecular alterations in correlation with their time to progression.

**Table 1:** *EGFR*-dependent and independent molecular alterations detected in cfDNA and/or CTCs of NSCLC patients treated with osimertinib at PD, ranked according to reducing the time of PD.

Patient ID	*EGFR* dependent		*EGFR* independent						PD (months)
	DNA mutations		DNA mutations *PIK3CA*, *KRAS G12C, BRAF V600E*	DNA methylation		Gene expression	IF	FISH analysis	
	cfDNA	CTCs	cfDNA	cfDNA	CTCs	CTCs	CTCs	CTCs	
**#9**	L858R	nd	nd	*RASSF1A*, *APC*, *WIF-1*	*APC*	nd* *	nd	nd	36.1
**#8**	nd	nd	nd	nd	nd	PIM-1*	nd	nd	31.3
**#24**	nd	nd	nd	nd	nd	PD-L1*	nd	nd	17.9
**#14**	nd	nd	nd	nd	nd	nd	nd	nd	15.9
**#2**	nd	nd	nd	nd	nd	nd	nd	nd	14.7
**#5**	nd	nd	nd	*BRMS1*, *SLFN11*	*SLFN11*	nd	nd	nd	13.8
**#12**	G719X	T790M	nd	*BRMS1*, *WIF-1*, *FOXA1*	*RASSF10*, *WIF-1*	PD-L1* PIM-1*	nd	nd	12.8
**#16**	nd	nd	PIK3CA* (H1047R)	*WIF-1*	nd	PD-L1*, AXL*	nd	nd	12.4
**#1**	T790M, del19, C797S	nd	PIK3CA* (E545K)	*RASSF1A*, *APC*, *RARb*	nd	* *nd	nd	nd	9.3
**#25**	nd	nd	nd	nd	*SLFN11*	PD-L1*, PIM-1*	nd	nd	9.2
**#23**	nd	nd	nd	nd	*APC*	PD-L1*, AXL*	nd	nd	8.9
**#21**	nd	nd	nd	nd	nd	PD-L1*	PD-L1*	nd	7.7
**#17**	del19	T790M	nd	nd	*SLFN11*	PD-L1*, AXL*, PIM-1*	PD-L1*	nd	7.4
**#29**	del19	nd	PIK3CA* (H1047R)	*WIF-1*	nd	nd	nd	nd	7
**#26**	nd	nd	nd	nd	nd	nd	nd	nd	6
**#18**	L858R	T790M	nd	*RASSF1A*, *BRMS1*, *APC*, *FOXA1*	nd	PD-L1*	nd	nd	5.9
**#7**	L858R	nd	nd	*WIF-1*	*APC*	nd	nd	nd	5.3
**#4**	nd	nd	nd	nd	nd	nd	PD-L1*	nd	3
**#30**	L858R	nd	PIK3CA* (H1047R)	nd	*SLFN11*	PD-L1*, PIM-1*	nd	nd	2.9
**#27**	L858R	nd	nd	nd	nd	nd	nd	nd	2.8
**#19**	del19	nd	nd	*APC*	nd	nd	nd	nd	2.8
**#15**	del19	nd	nd	nd	nd	PIM-1*	nd	nd	2.8
**#6**	T790M, L858R	nd	nd	*RASSF1A*, *APC*	*APC*	PIM-1*	nd	*HER2* amplification*	2.6
**#11**	nd	nd	nd	nd	nd	PD-L1*, PIM-1*	nd	* *nd	2.5
**#20**	del19	nd	nd	*RASSF1A*, *APC*, *RARb*	*SLFN11*	PIM-1*	* *nd	* *nd	2.3
**#10**	G719X, S768I	G719X, S768I	nd	*RASSF1A*	nd	AXL*	PD-L1*	* *nd	1.6
**#28**	del19	nd	nd	*BRMS1*, *SLFN11*, *APC*	nd	nd	* *nd	* *nd	1.4

nd, not detected. *: Therapeutic targets.

## 4 Discussion

Comprehensive analysis including alternative molecular alterations that may occur at resistance to osimertinib is important for guiding subsequent treatments, especially for those patients who relapse early. The present study highlights the potential of combining liquid biopsy analytes to elucidate NSCLC molecular heterogeneity in patients under osimertinib treatment. Comprehensive liquid biopsy analysis for DNA mutations, DNA methylation markers, gene expression and chromosomal level in both plasma-cfDNA and CTCs revealed druggable molecular alterations and resistance mechanisms that may occur under selective therapy pressure. Discrepancies observed between plasma-cfDNA and paired CTCs are representing tumor heterogeneity, a well-known characteristic of NSCLC. Our results indicate that complementary information derived from plasma-cfDNA and CTC analysis proved to be very informative for treatment monitoring of NSCLC patients.

There are only a few direct comparison studies between CTC and plasma-cfDNA up to now, that use identical same blood draws and identical methodologies to detect mutations, DNA methylation markers, or gene expression not only in NSCLC (49, 69, 70, 79) but also in other types of cancer (59–61, 64). These few studies have shown that there are significant discrepancies in information derived from CTC and plasma-cfDNA material, especially when very low target concentrations are present in the sample. Currently, ongoing clinical trials include parallel analysis of CTCs and ctDNA in many types of cancer to test if this dual analysis is more sensitive for disease monitoring (80).

*EGFR* mutation analysis in the context of the current comprehensive study confirmed the significance of combined liquid biopsy analysis. Our previous results (70), have shown that T790M resistance mutation was detected at baseline in gDNA isolated from CTC but not in the corresponding plasma cfDNA (P#11) indicating that this may be a case of subclonal T790M, that is potent to lead to early PD. This finding is consistent with data from the AURA3 phase III trial (22). EGFR T790M mutation was detected at PD only in gDNA samples isolated from the CTCs of three patients but not in the corresponding plasma-cfDNA. There was only one case (P#1) of acquired resistance that was due to *EGFR* p.C797S mutation, which is one of the most frequent resistance mechanisms to second-line osimertinib treatment (81). Importantly, C797S mutation was detected *in cis* configuration with T790M, suggesting that the administration of brigatinib with an anti-EGFR antibody would be beneficial to the patient (82, 83) or fourth-generation EGFR TKIs (26). BLU-945 is another *EGFR* inhibitor, and the benefits when combined with osimertinib are being investigated in the phase I clinical trial SYMPHONY (NCT04862780). The rechallenge with first or second EGFR TKIs to NSCLC patients who acquired the C797S resistance mutation during osimertinib treatment has also been proposed, delaying the use of platinum-based chemotherapy since there is no approved targeted therapeutic strategies in this setting, so far (84).

The presence of the *BRAF* V600E mutation may limit the activity of EGFR TKIs in EGFRm NSCLC, as this was previously shown (85). *BRAF* V600E mutation as a resistance mechanism to osimertinib has been identified in 3% of NSCLC patients positive for *EGFR* mutations, irrelevant of the presence of a T790M mutation (25, 29, 86). Concomitant detection of *BRAF* and *EGFR* mutations has been reported in a few cases (87, 88), and in these cases, patients progressed more rapidly upon EGFR TKIs (89, 90). For this reason, different combination therapies have been proposed for patients carrying mutations in *BRAF* in EGFRm lung cancer (91–94). In this study, we did not detect any sample positive for *BRAF* V600E.

*KRAS* G12C was not detected in the samples analyzed. Although alterations in the RAS-MAPK pathway have been shown to lead to osimertinib resistance, cases reporting the presence of *KRAS* G12C mutation are limited (26). However, there is only a recent case report study of acquired *KRAS* G12C mutations during first-line osimertinib resistance which showed the effective and well-tolerated combination of osimertinib and sotorasib (95). Sotorasib which has been recently approved by the FDA for patients with locally advanced or metastatic NSCLC, carrying *KRAS* G12C mutations could be an option for those who progress to osimertinib.

*PIK3CA* mutations were detected only in plasma-cfDNA but not in corresponding CTCs*. PIK3CA* mutations have been detected in a frequency of 4%-14%, as a resistance mechanism against second-line osimertinib treatment. The most common are the *PIK3CA* hotspot mutations H1047R, E545K, and E542K (81, 96, 97). Results from the AURA3 trial have shown that *PIK3CA* alterations were detected more frequently in the T790M positive NSCLC patients (98). *PIK3CA* concurrent mutations confer resistance to osimertinib as confirmed with *in vitro* experiments (22). Based on previously reported evidence, these patients could have been treated with appropriate combination therapies against the PIK3/AKT/mTOR pathway (22, 99). Han et al. reported that both *PIK3CG* (L468M) and *PIK3CA* (H1047R) mutations could induce osimertinib resistance through PI3K/Akt/mTOR pathway-dependent mechanisms. They proposed the administration of aspirin which could effectively reverse *in vitro* and *in vivo* osimertinib resistance as a treatment strategy for NSCLC patients who develop these mutations (100).

In our comprehensive analysis, we used FISH analysis to assess the presence of c*MET* and *HER2* amplification in CTCs enriched with the microfluidic platform Parsortix. To the best of our knowledge, this is the first study that detects these two gene alterations in NSCLC patients before and after osimertinib treatment using FISH. In our study, *MET* amplification was detected in the CTCs of two patients (P#8, P#25) before treatment with osimertinib. *MET* amplification in NSCLC may be a primary oncogenic alteration or may arise as a secondary driver resistance mechanism to EGFR TKI treatment through the activation of downstream signaling pathways, like MAPK or PI3K-Akt (98, 101). Up to date, the standard of care for EGFRm patients with *MET* alterations is platinum-based chemotherapy with limited efficacy. However, recent data from the TATTON trial have shown that combination therapy of osimertinib with the *MET* inhibitor, savolitinib, presented acceptable tolerability and clinical activity (102). Consequently, patients in our study positive for *MET* amplification at baseline could have benefited from such a combinatorial therapeutic strategy. Currently, the ongoing SAVANNAH study aims to examine the efficacy of this treatment combination in patients with MET-mediated who progressed following treatment with osimertinib whereas the SAFFRON study (NCT05261399) is designed to assess savolitinib in combination with osimertinib versus platinum-based chemotherapy in the same group of patients (26).

Based on our results, *HER2* amplification was detected in the CTCs of three patients before treatment with osimertinib (P#1, #8, #25) and in one patient at PD (P#6). *HER2* gene amplification is another common osimertinib resistance mechanism that leads to the activation of HER2 signaling followed by the downstream activation of the PI3K-Akt pathway. Results from the AURA3 trial demonstrated that 5% of patients who progressed on second-line osimertinib treatment had *HER2* amplification (98). TRAEMOS is the first trial testing the combination of trastuzumab-emtansine and osimertinib to target *HER2*-mediated resistance in patients with EGFRm NSCLC. Despite the favorable safety profile, this combination revealed limited efficacy to patients (103).

It is now well known that dominant tumor cancer cells are subjected to epigenetic modifications and switch to drug-resistant cancer cells in various types of cancer (104–106). DNA methylation provides useful insights into lung cancer development and is correlated with early detection, prognosis, and prediction of response to specific treatments (107). Liquid biopsy is a very powerful tool for identifying circulating DNA methylation markers that could be of clinical importance (107, 108). Concerning the role of methylation in resistance to EGFR ΤΚΙ therapy, there are now studies performed in lung cancer cell lines or primary tissues showing that epigenetic modifications negatively affect EGFR TKI treatment outcome and that their combination with epigenetic drugs could be very promising (109–115). Intriguingly, a methylation-associated mechanism behind the acquisition of T790M mutation was previously described (113). Recent advances in DNA methylation modifications linked to TKI resistance mechanisms in EGFRm patients have been previously reported (108). However, only a few studies focused on DNA methylation in ctDNA or CTCs of NSCLC patients receiving osimertinib (69, 116, 117).

We have previously reported results on DNA methylation of *RASSF1A, RASSF10, APC, WIF-1, BRMS1, SLFN11, RARβ, SHISA3*, and *FOXA1* in plasma-cfDNA and paired CTCs of NSCLC patients during osimertinib therapy. There was a low concordance of DNA methylation markers in CTCs and cfDNA, indicating the importance of complementary information obtained through parallel CTCs and cfDNA analysis. A predictive role of DNA methylation as a potential resistance mechanism was shown in this study where patients with at least one methylated marker in liquid biopsy samples at PD eventually progressed earlier than those negative for methylation (69).

CTC analysis at the gene expression level provides important information on tumor heterogeneity and can reveal differential gene expressions related to metastasis or treatment sensitivity and resistance (72). The first gene expression study in CTCs of NSCLC patients during osimertinib treatment revealed heterogeneous patterns of gene expression of epithelial, mesenchymal/EMT, and stem cell markers among patients. A potential role of EMT was shown based on the high rates of vimentin-positive CTCs detected at all time points during osimertinib treatment (118).

The increased number of NSCLC patients at PD harboring CTCs positive in *PD-L1* suggests a theoretical background for immune checkpoint inhibition (ICI) therapy in EGFRm NSCLC patients resistant to osimertinib (73). However, the combination of data from IF and RT-qPCR for the presence of PD-L1 positive CTCs in matched samples revealed high detection rates both at baseline and at PD. Immunotherapy treatments for EGFRm NSCLC are still a big challenge since to date numerous studies have shown the confined efficacy of immunotherapy either as monotherapy or in combination with chemotherapy (118).

Our study was the first to evaluate *AXL* gene expression levels in CTCs of patients during osimertinib therapy (73). Several preclinical studies on *AXL* inhibition suggest this approach as a new additional tool for personalized therapy of NSCLC patients with *EGFR* mutations, since these patients may benefit from *AXL* inhibitors (119–122). EGFRm NSCLC patients harboring high levels of *AXL* expression had significantly shorter PFS and OS after ICI-based therapy (123).

Herein, we detected *PIM-1* expression in CTCs of EGFRm NSCLC patients before osimertinib and at PD, suggesting that concurrent use of *PIM-1* inhibitors with osimertinib could be a possible therapeutic strategy. The synergistic effects of *PIM* inhibitor in combination with osimertinib acting through the inhibition of oncogenic signaling pathways have previously been reported (124, 125). *EGFR* signaling is indirectly affected by *PIM-1* suggesting that *PIM-1* inhibition can improve patient’s outcomes (124, 126).

The current study highlights the potential of analyzing both CTC and ctDNA derived from a single blood draw to identify molecular biomarkers clinically significant for the patient’s outcome or alternative treatment approaches upon osimertinib treatment. We would like to point out that in our study we have included different methodologies for the identification of molecular alterations at the DNA, RNA, and epigenetic level. According to recent guidelines for NSCLC, NGS approaches are the most suitable for the identification of multiple molecular alterations in LB samples but only at the DNA level (41, 127, 128). Commercially available technologies such as nanopore DNA sequencing offer now analysis of whole genome sequencing and the identification of DNA methylation aberrations, simultaneously (129–131). However, such a commercially available integrated approach before and after treatment with osimertinib has not been performed so far.

Treatment of NSCLC patients with osimertinib is very challenging if we consider the high molecular heterogeneity of this disease and also the clonal evolution that arises through selective therapy pressure. These two significant factors compose the wide spectrum of resistance mechanisms that claim early identification and proper therapeutic interventions. In the present study, it was clearly highlighted the potential of comprehensive liquid biopsy analysis to efficiently represent the heterogeneous molecular landscape and provide prominent information on subsequent treatments for NSCLC patients based on the druggable molecular alterations found at PD. Epigenetic alterations give additional information to DNA mutation analysis to identify patients who are unlikely to benefit from EGFR TKI therapy. Studies have shown that targeting epigenetic alterations might be a therapeutic intervention to reverse EGFR TKI resistance. Complementary information obtained from cfDNA and CTC analysis is of utmost importance during the management of NSCLC patients.

## Data Availability

The raw data supporting the conclusions of this article will be made available by the authors, without undue reservation.
